# Specific Features of Mandible Structure and Elemental Composition in the Polyphagous Amphipod *Acanthogammarus grewingkii* Endemic to Lake Baikal

**DOI:** 10.1371/journal.pone.0043073

**Published:** 2012-08-10

**Authors:** Irina V. Mekhanikova, Dmitry S. Andreev, Olga Yu. Belozerova, Yuri L. Mikhlin, Sergey V. Lipko, Igor V. Klimenkov, Vladlen V. Akimov, Valeriy F. Kargin, Yelena V. Mazurova, Vladimir L. Tauson, Yelena V. Likhoshway

**Affiliations:** 1 Limnological Institute of the Siberian Branch of the Russian Academy of Sciences, Irkutsk, Russia; 2 A.P. Vinogradov Institute of Geochemistry, Siberian Branch, Russian Academy of Sciences, Irkutsk, Russia; 3 Institute of Chemistry and Chemical Technology, Siberian Branch, Russian Academy of Sciences, Krasnoyarsk, Russia; Federal University of Rio de Janeiro, Brazil

## Abstract

**Background:**

In crustaceans, several mechanisms provide for the mechanical strength of the cuticular “tools” (dactyli, claws, jaws), which serve to catch and crush food objects. Studies on the mandibles of the endemic Baikal amphipod *Acanthogammarus grewingkii* by means of electron microscopy and elemental analysis have revealed specific structural features of these mouthparts.

**Methodology:**

The fine structure of the mandible has been studied by means of SEM, TEM, and AFM; methods used to analyze its elemental and phase composition include XEPMA, XPS, SEM-EDS analysis, and XRD.

**Conclusion:**

Functional adaptations of the mandible in *A. grewingkii* provide for the optimum combination of mechanical hardness and fracture resistance, which is achieved due to a complex structure and composition of its cutting parts. Teeth of the mandible are covered by a thin layer of silica (10–20 µm). Their epicuticle is characterized by a high density, consists of three layers, and increases in thickness toward the tooth apex. The epicuticle is enriched with Br, while the concentrations of Ca and P reach the peak values in the softer internal tissues of the teeth. These data broaden the view of the diversity of adaptation mechanisms providing for the strengthening of cuticular “tools” in crustaceans.

## Introduction

The mouthparts of crustaceans are head appendages that have undergone a long evolution into complex, highly specialized cuticular structures effective in catching and crushing various food objects. Recent scanning and transmission electron microscopy studies on six species of endemic Baikal amphipods (Crustacea, Malacostraca) differing in habitat, mode of life, and diet have provided evidence for the relationship between the morphology of the mandible (MD) and the feeding strategy of the species [1].

The greatest load in crushing hard food is borne by the MD cutting edge, or incisor (IN), which therefore must be hard, strong, and resistant to wear and cracking. A widespread mechanism imparting the requisite properties to the exoskeleton of crustaceans consists in its mineralization, e.g., reinforcement of the cuticle with calcium compounds, which improve its wear resistance [2]–[4]. An illustrative example of mineralization is provided by marine copepods (Crustacea, Entomostraca), in which the teeth on the mandibular gnathobase have siliceous crowns (opal teeth) that reach 7 µm in thickness at the tooth apex [5]. These evolutionarily acquired structures serve to crush hard food objects such as siliceous shells (frustules) of diatoms, which, in turn, have evolved so as to provide effective mechanical protection to algal cells against being eaten away by consumers [6]. Another widespread mechanism for strengthening the cuticular “tools” of invertebrates is sclerotization, or cross-linking of linear protein and polysaccharide molecules into three-dimensional structures. The cross-linking may be a result of reaction with organic molecules (e.g., quinones) or transition metals (e.g., Zn, Cu, Mn, Fe) [7]–[9], or of chemical modification of the cuticle with the involvement of halogens (Br, Cl, I) [10], [11].

**Figure 1:**
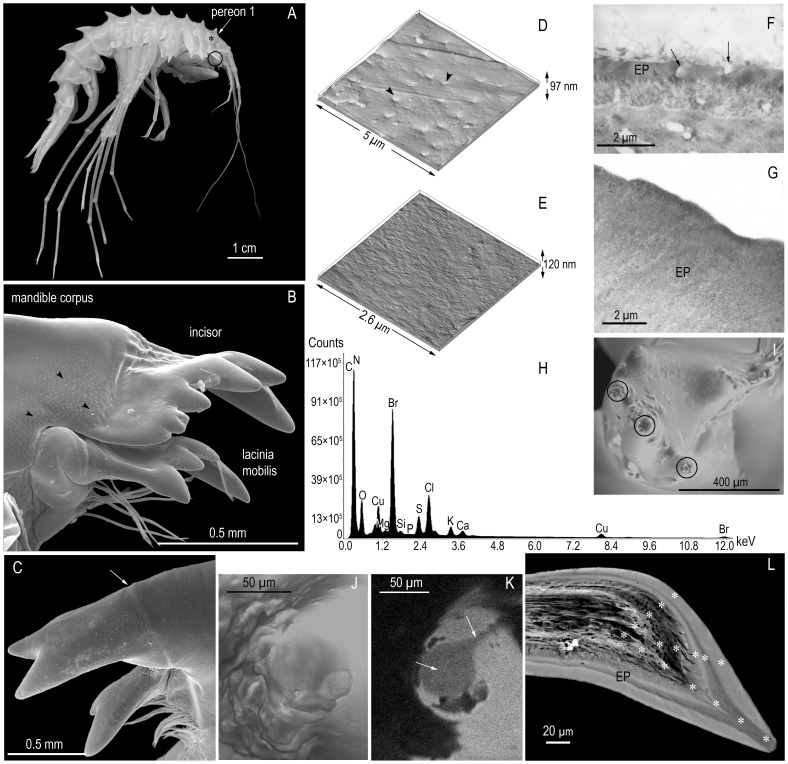
Mandible structure and elemental spectrum of the tooth area. (**A**) *Acanthogammarus grewingkii,* external view. The star indicates the centrolateral part of P1 where the AFM and TEM images were taken and XEPMA measurements of elemental composition were made; the circle indicates the location of MD. (**B**) The distal part of the left MD (SEM). Arrowheads indicate pores. (**C**) The first IN teeth on the left MD (SEM). The arrow indicates the boundary between the cutting edge and corpus of MD. (**D**) The cuticular surface of P1 (AFM). Arrowheads indicate pores. (**E**) The surface of an IN tooth with signs of wear (AFM). (**F**) An ultrathin section through the EP of P1 (TEM). Arrows indicate pores. (**G**) An ultrathin section through the external EP of an IN tooth (TEM). (**H**) X-Ray elemental spectrum of the mapped IN tooth area (SEM-EDS). (**I**) Apical parts of IN teeth on the right MD, top view. Circles indicate Br-depleted areas (SEM-EDS). (**J**) The apical part of IN tooth with traces of wearing, top view (SEM). (**K**) X-Ray map of Br distribution in the apical part of an IN tooth. Arrows indicate Br-depleted areas (SEM-EDS). (**L**) The layered structure of EP in a section through the IN tooth apex (image in back-scattered electrons in a Superprobe JXA-8200 scanning electron microscope). Stars indicate points of elemental composition measurements.

Among Baikal amphipods, the largest and highly abundant species *Acanthogammarus grewingkii* has been chosen for this study (Figure 1A). This species inhabits the deep zone of the lake (from 300–400 to 1,300 m) under exclusively stable conditions [12]. Diurnal and seasonal fluctuations of physical and chemical parameters in the ultrafresh oligotrophic Lake Baikal occurs only in the uppermost water layer up to 250 m. Below this layer and up to maximal depths, the water temperature and pressure are constant, saturation with oxygen is not less than 70–80% even at maximal depths, the distribution of major ions is uniform within all layers and does not subject to seasonal variations, and the water lacks calcium and microelements [13]–[15]. The polyphagous amphipod *A. grewingkii* feeds on any food available, including organisms with hard shells, such as diatoms and crustaceans, breaking them into fragments. Planktonic diatoms, including the thick-shelled species *Aulacoseira baicalensis* [16], play a significant role in its feeding [1], [17] in the years of its mass growth.

The purpose of this work was to study the structure and elemental composition of cutting edges of the mandible of *A. grewingkii* inhabiting the low mineralized environment of the oligotrophic Lake Baikal, and to reveal adaptation of the mandible to breaking of food objects with hard shells. The scientific literature lacks data on thin structure of the mandible of amphipods (as well as representatives of other orders of Malacostraca), on its element composition and distribution of elements in different mandible layers.

In this work, several analytical methods were used to reveal specific features of the structure and composition of the MD cutting edge. Results showed that mandibular tissues structurally differ from the cuticle of the first thoracic segment, or pereon 1 (P1), and that the epicuticle (EP) of mandibular incisor (IN) teeth consists of several layers; the distribution of chemical elements in different tissue layers of IN teeth has been analyzed.

## Materials and Methods

### Sampling

Amphipods were sampled with trawls in different areas of Lake Baikal during the years 1996, 2007, 2009, 2011. Adult individuals selected for the study (Figure 1A) were fixed in 4% formalin or 75% ethanol (for TEM they were frozen in liquid nitrogen) and dissected with microsurgical scissors under a binocular microscope. In addition to MD (Figure 1B), samples of P1 cuticle from its centrolateral part were taken for comparative analysis by certain methods (Figure 1A).

### Scanning Electron Microscopy (SEM)

The MD fixed with 4% formalin were dehydrated in an ethanol series, dried, glued onto aluminum stubs with carbonic adhesive tape, vacuum-coated with gold in a Balzers SCD 004 sputter coater, and examined under a Philips SEM 525M scanning electron microscope. The conditions for analysis: voltage acquisition 28.9 kV, working distance 10 mm. Fourteen mandibles were analyzed.

### Scanning Atomic Force Microscopy (AFM)

Samples of MD and P1 cuticle were fixed in 75% ethanol, dried, glued onto stubs, and analyzed by contact-mode AFM in an SMM-2000 scanning multimicroscope (Russia) using silicon nitride cantilever tips with a curvature radius of 30 nm (Veeco Instruments, United States). Optimal conditions for analysis were as follows: applied force, 300 nN; scan step, 6.1 Å; scan rate, 15 µm s^−^^1^. The maximum resolution was about 2.5 nm in the XY plane and 1.13 nm along the Z axis. Two teeth of one mandible and a cuticle fragment of P1 were analyzed. Twenty pores were measured and their density was determined per unit of surface area of P1. Statistical analyses were conducted using STATISTICA-6 software package for Windows 7.0.

### Transmission Electron Microscopy (TEM)

The material (MD and P1 cuticle) was frozen in liquid nitrogen, then after thawing the samples were fixed with 2.5% glutaraldehyde solution in 0.1 M phosphate buffer, postfixed in 2% OsO_4_ solution, dehydrated in an ethanol series, and embedded in epoxy resin (Araldite 502 Kit SPI, United States). Semithin sections (1 µm) made with an Ultracut R ultratome (Leica, Austria) were examined under an Axiovert 2000 microscope (Carl Zeiss, Germany) to select areas of interest. Ultrathin sections of these (50–60 nm) were studied under a Leo 906E transmission electron microscope (Carl Zeiss). Microscopic images taken with a SIS MegaView II digital camera were processed using the MegaVision program package (Soft Imaging System GmbH, Germany). Twelve mandibles and 4 cuticle fragments of P1 were analyzed.

### Scanning Electron Microscopy with Energy-Dispersive Analysis (SEM-EDS)

For surface analysis, MD samples fixed in 75% ethanol were dried and mounted on carbon-coated polystyrene film or TEM grids. Their internal structure was studied in the same polished sections that were used for local elemental analysis by the XEPMA method (see below). Element mapping was performed in a Quanta 200 scanning electron microscope (FEI Company, United States) with a Genesis XM 2 60 energy-dispersive X-ray detector (EDAX, United States). The samples were scanned either in high vacuum (in this case, they were sputtered with carbon in a JEOL JEE-4B vacuum evaporator) or in a nitrogen atmosphere at a pressure of 60 Pa under the following conditions: acceleration voltage, 25 kV; current, 100 µA; working distance, ∼10 mm. The beam dwell time was 7 hours on average and resolution 256×200 pixels, the energy spectrum was recorded in the range of 0–20 keV. Fourteen mandibles were analyzed: 4 on the polished sections and 10 entire mandibles.

### X-Ray Electron Probe Microanalysis (XEPMA)

The MD fixed in 4% formalin were dried, dehydrated, impregnated with Buehler EPO-Thin resin, and cleaved in the sagittal plane; thereafter, they were embedded in EDP-5 resin to prepare polished sections, which were sputter-coated with carbon (20–30 nm). The chemical composition of the samples was studied using a Superprobe JXA-8200 microanalyzer (JEOL, Japan) with an energy-dispersive spectrometer (EDS) at an accelerating voltage of 20 kV, probe current of 2–4 nA, beam diameter of 1 µm, and spectrum collection time of 60 s. The spectra were processed by the Semiquantitative Analysis program of software for EDS Superprobe JXA-8200 device. Four mandibles were used for preparation of polished sections in epoxy resin, elemental composition was measured in 35 points. Statistical analyses were conducted using STATISTICA-6 software package for Windows 7.0.

### X-Ray Photoelectron Spectroscopy (XPS)

The MD and P1 cuticle samples fixed in 4% formalin were dried and mounted on sample holder with double-faced conducting carbon tape. Photoelectron spectra were recorded on a SPECS spectrometer (Germany) equipped with a PHOIBOS 150 MCD9 hemispherical energy analyzer at a pass energy of 8–10 eV for high-resolution spectra or 20 eV for survey spectra using nonmonochromatized Mg Kα irradiation (1253.6 eV). This method provides information on the average elemental composition of the surface layer (approximately 1–2 nm thick) in an area of about 2–3 mm^2^. Sample etching with Ar^+^ ions was performed using a scanning ion source operated at an accelerating voltage of 5 kV and emission Ar^+^ current of 30 µA; the rate of etching was about 1–2 nm min^−^^1^. Eighteen mandibles and 9 cuticle fragments were analyzed.

### X-Ray Diffractometry (XRD)

Analysis of MD samples dried after fixation in 4% formalin was performed in a DRON-2.0 X-ray diffractometer (Russia) under the following conditions: Cu Kα source, 32 kV, 32 mA, angular range 5–70° (2θ). The spectra were identified with reference to the database of the Joint Committee on Powder Diffraction Standards (JCPDS), International Center for Diffraction Data. The bulk composition of MD was determined using aliquots of a pooled sample of these mouthparts (without palps and muscle tissues) from several tens of individuals.

## Results

According to data of scanning (SEM), atomic force (AFM), and transmission electron microscopy (TEM), the cuticle of *A. grewingkii* on P1, MD corpus, and MD cutting edge differs in structure (Figures 1B–1G). Within cuticular polygons on P1, which in this species are bordered at the perimeter by fused microtrichs [18], AFM analysis revealed pores 160–590 nm in diameter, average size of pores being 350±26 nm (larger pores are sometimes recorded – up to 700 nm); their density is approximately 1 pore/µm^−2^. The polygons have a surface roughness (by height) of 11 nm (Figure 1D). As shown by TEM of ultrathin sections, the P1 cuticle (including EP) is permeated by pore canals with surface openings (pores) (Figure 1F). The MD corpus is also covered with pores (Figure 1B), whereas its cutting edge, delimited by a distinct boundary (Figure 1C), has a smooth surface: its roughness in undamaged areas is only 5 nm according to AFM data (Figure 1E). In ultrathin sections, the EP of IN teeth is dense and homogeneous, without pore canals and pores (Figure 1G). During SEM analysis, only one specimen was found to contain three pores on the distal surface of an IN tooth, which were larger than pores in the MD corpus and P1 cuticle.

SEM-EDS analysis revealed the presence of Br on the surface of IN teeth, except for their apical parts where Br-depleted spots 20–30 µm in diameter were found (Figure 1I). In addition to Br, elements detected on the tooth surface include C, N, O, Ca, Mg, Si, S, P, and K (the Cu peak comes from the specimen supporting grid) (Figure 1H). Depending on the degree of IN tooth wear, its surface deformation (Figure 1J) and cracking of the Br-rich layer (Figure 1K) could be observed.

The investigation by XEPMA (backscatter SEM) of polished sections of *A. grewingkii* IN teeth in back-scattered electrons using JXA-8200 scanning (raster) electron microscope showed that they have a stratified (layered) structure (Figure 1L). The three external homogeneous layers differing in electron scattering intensity are apparently epicuticular layers (EP1, EP2, EP3). The thickness of EP is slightly greater on the concave than on the convex edge of the tooth, reaching a peak of 50–90 µm at its apex. In specimens where the section passed through the tip of the tooth, it could be seen that the middle layer EP2 increases in thickness toward the tooth apex, where it emerges on the surface (Figure 1L).

**Figure 2:**
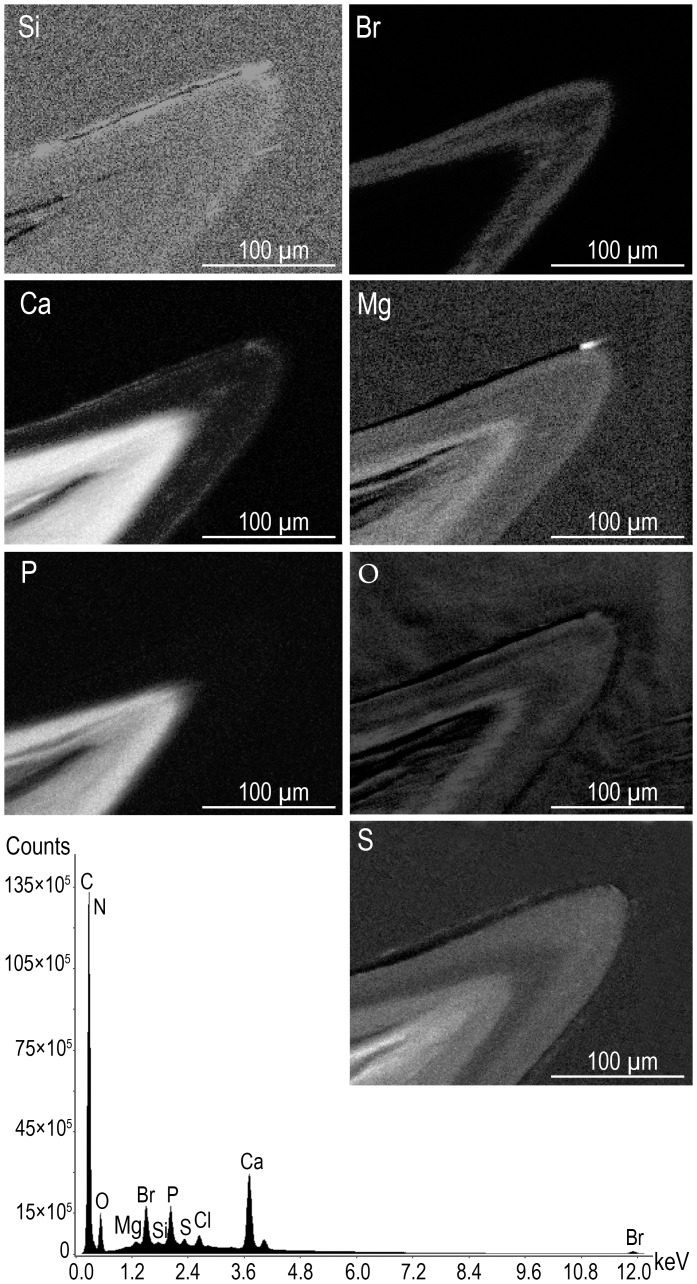
X-Ray elemental spectrum of a polished section of IN and X-Ray element distribution maps (SEM-EDS).

SEM-EDS analysis of such sections revealed peaks for a number of elements (C, N, O, Br, Ca, Mg, Si, S, P) and allowed their distribution to be mapped (Figure 2). It was found that Si forms a thin (about 10–20 µm) film on the tooth surface, which is underlain by Br-containing layers. The boundaries of these layers coincide with those of the three EP layers revealed by scanning electron microscope in back-scattered electrons using Superprobe JXA-8200 microanalyzer (XEPMA) (Figure 1L), and the middle EP2 layer (emerging on the surface at the tooth apex) is relatively poor in Br. Calcium is present in the surface EP1 layer, but the bulk of it concentrates in softer tooth tissues, under brominated EP layers, where it colocalizes with P (which has not been detected in EP). Magnesium is contained both in the surface EP1 layer (∼10 µm) and under EP, and is colocalized with O. Sulfur is detected in EP (mainly in EP1 and EP2) as well as in the softer tissue under EP, where it colocalizes with Ca and P (Figure 2).

Local elemental analysis by the XEPMA method in polished sections of IN teeth confirmed the results of mapping. It was shown that Br in significant amounts is contained in all three EP layers, where its concentration ranges from 11 to 17 wt.%, but is absent in underlying tissues. Conversely, the concentration of Ca reaches a peak of about 7 wt.% in the internal tooth tissues, which also contain about 2 wt.% of P (Table 1). Different layers of the MD cutting edge in the sections consist mainly of organic matter rich in C and O, with the total contents of organic elements (C, O, and N) varying from 81 to 89 wt.% between the specimens (Table 1). The spectra of analytical lines of determined elements obtained applying energy-dispersive spectrometer (EDS) also show the presence of background amounts of Mg, S, K, Na, F and some other elements.

**Table 1:** Elemental composition (wt.%) of IN teeth in *Acanthogammarus grewingkii* (XEPMA).

	EP1	EP2	EP3	Internal tooth tissues
C, O, N	81.7	87.2	81.5	89.2
Other elements	18.3	12.8	18.5	10.8
Including:				
Ca	1.3±0.5	0.6±0.2	3.0±1.0	6.6±1.0
Br	16.7±2.0	11.3±1.7	14.6±1.9	0
P	0	0	0	2.0±0.3
Al	0.2±0.2	0.3±0.3	0.9±0.8	0.2±0.05
Cl	0.1±0.1	0.6±0.5	0	1.0±0.2
	*n* = 7	*n* = 8	*n* = 6	*n* = 24

Note: F, Mg, S, K, Na, Si, Ti, Cr, Fe, Zn, and Sr were detected only at a background level in some layers of IN teeth; *n* is the number of measurements.

**Figure 3:**
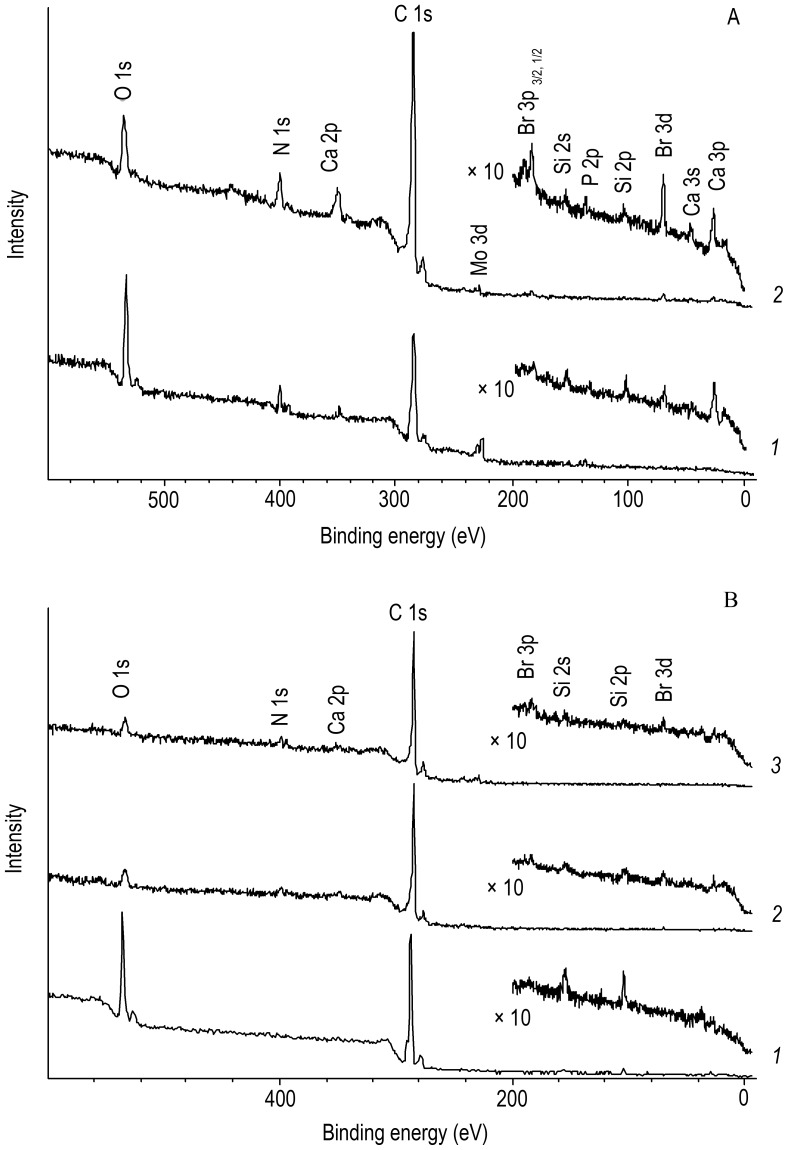
X-Ray photoelectron spectra in the range of binding energies of up to 500 eV. (**A**) the P1 cuticle and (**B**) the MD surface (*1*) before Ar^+^ etching and after the etching for (*2*) 2 min and (*3*) 20 min.

The results of XPS analysis of the P1 cuticle show that, in addition to C and O, its surface is enriched with N and Ca (typical atomic concentrations 6–7 and 1–3 at.%, respectively) and also contains lower concentrations of Br, P, Si, and S (Figure 3A). After the removal of 1–2 nm surface layer by etching with Ar^+^ ions, the intensity of O 1s lines decreases, while that of N, Ca, Br, and P lines increases, indicating corresponding changes in the concentrations of these elements (Figure 3A, diagram *2*). Weak Mo signals apparently come from the specimen holder.

The MD surface, which is also rich in C and O, contains 2.5–3 at.% of Si; in addition, there are small amounts of N, Br, P, and Ca (Figure 3B). Judging from the Si 2p binding energy ∼102.3 eV, Si has the oxidation degree +4 and is bound to oxygen, apparently in the form of dioxide. The intensity of Si lines slightly drops after Ar^+^ etching, but they still remain even when the surface layer has been removed to a depth of 15–20 nm (Figure 3B, diagrams *2, 3*). Upon longer etching, the lines of Ca, N, and Br appear in the spectrum or increase in intensity. The concentrations of Ca and N are low and change only slightly. Br 3d lines may be fitted with two components: one has a binding energy of 68.5 eV (ionic bonding as in NaBr, KBr, etc.), and the other, at about 70.4 eV, is characteristic of Br bound to carbon (covalent bonding). Bromide ions seem to concentrate mainly on the surface, while covalently bound bromine occurs in deeper layers, but these require further examination.

**Figure 4:**
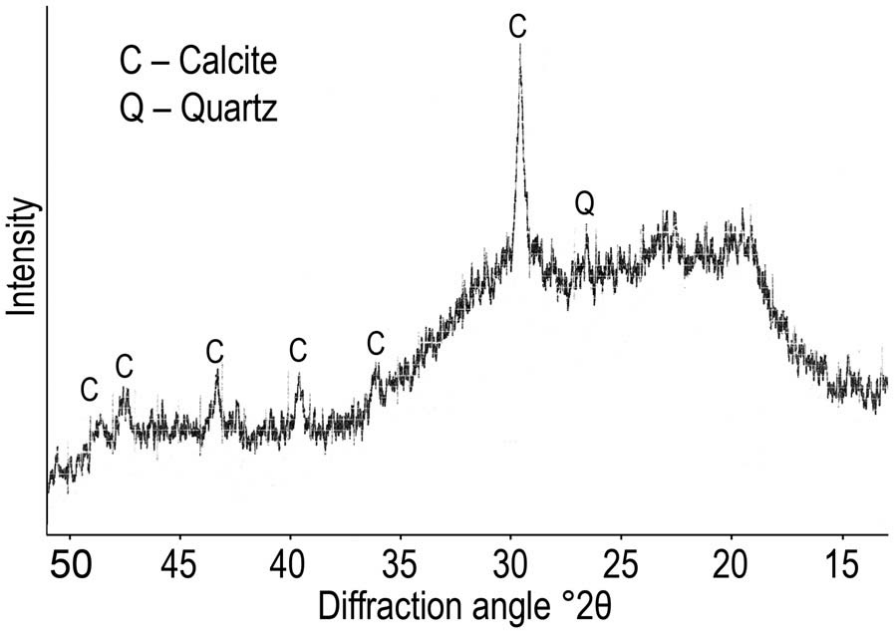
X-Ray diffraction pattern of calcite and quartz in the MD of *A. grewingkii* (XRD).

Analysis by XRD confirmed that pooled MD samples contained crystalline CaCO_3_ and small amounts of alpha quartz (α-SiO_2_) (Figure 4).

## Discussion

Differences in the structure of the cuticle between the body parts of crustaceans are explained by their different functions. The cuticle of the MD corpus and P1 in *A. grewingkii* has numerous pores, which are the external openings of transcuticular pore canals (Figures 1B, D, F). The presence of pore canals and cavities in the cuticle reduces its weight and facilitates matter exchange between the body and the external environment. In amphipods, the pore canals and cavities in EP may account for as much as up to 30% of the total carapace volume [19]. The cutting edge is separated from the MD corpus by a groove and differs from it in surface structure, in particular, the absence of pores and microtrichs and higher density (Figures 1B, C, E, G). A few large pores revealed by SEM in one IN tooth specimen are probably the openings of tegumental glands. Such pores have been found at the tips of dactyli on the walking legs of *Cancer pagurus* crabs, and there appears are dependence between the number of these pores and the intensity of the amber coloration of the dactyli in this species [20].

The layered structure of EP of IN and its increasing thickness in the apical part are important factors in the MD functioning. The apical parts of IN teeth bear the greatest load in crushing hard food, and, as shown in previous studies on different species, it is these parts that become worn down during intermolt periods in polyphages and spongiophiles [1]. In *A. grewingkii,* however, individuals with broken IN teeth occur very rarely. The middle epicuticular layer (EP2) emerges on the surface at the tooth apex (Figures 1I, K, L), which can sometimes be observed even under a light microscope [1]. Owing to the great thickness of EP in the apical parts of teeth (up to 90 µm), they remain functional even when worn down. This is very important for crustaceans, since molting – the total replacement of the exoskeleton, including mouthparts – is a very energy-expensive process.

The contents of chemical elements in the IN teeth of *A. grewingkii* is specific for each layer. According to the results of element mapping, Si forms a 20-µm layer on the EP surface (Figure 2). Ionic etching has shown that the Si line does not disappear from the spectrum when this layer is removed (Figure 3). The probable function of this siliceous film is to improve both the hardness and adhesive properties of the IN teeth surface. It appears that its effect on the mechanical properties of the apical parts of IN teeth manifests itself mainly during the early postmolt period, when they are not yet worn down. Unlike in marine copepods, which have opal teeth on the mandibular gnathobase [5], the siliceous film in *A. grewingkii* rests on brominated EP layers and has approximately equal thickness on the lateral and apical parts of teeth.

The high hardness of cuticular tools can be achieved due to mineralization involving the deposition of calcium salts [2], [4], with integuments mineralized to a lesser degree remaining more elastic [21]. In the cuticles of most crustacean species, Ca is contained as a crystalline salt (mainly calcite), which may also occur together with its amorphous form [2], [22]. According to XRD data, the crystalline phase of the total MD in *A. grewingkii* consists mainly of calcite (Figure 4). Other crystalline salts have not been detected (probably because of the small size of the aliquot used for analysis), but the results of element mapping show that their presence is possible. Thus, the content of Ca increases upon transition from the hard EP layers to underlying softer tissues, where this element colocalizes with P (Figure 2); therefore, Ca phosphate may also contribute to the strength and architecture of these tissues. Throughout the IN tooth section, Ca has proved to occur together with Mg. This fact is noteworthy, because the Ca/Mg ratio is relevant to the maintenance of the optimum balance of hardness and flexibility in cuticular structures [23], [24].

Unlike the highly mineralized teeth of vertebrates, the mouthparts of many invertebrates consist of a biomacromolecular scaffold reinforced with relatively small amounts of mineral substances [25], [26]. As shown in many groups of invertebrates, the main role in the hardening of noncalcified cuticular structures, including specialized cuticular “tools”, is played by sclerotization (in particular, bromine-dependent sclerotization). Sclerotized tissues are more flexible, though less hard than calcified tissues [11], [27]. Comparisons with synthetic materials have shown that the bromine-contained substance of the cuticle of crabs is harder and stronger than polycarbonate, acrylic, and nylon 6 [11]. Judging from the binding energy values (Figure 3), Br in the cuticular structures of *A. grewingkii* is contained in organic compounds. The results of element mapping, confirmed by XEPMA analysis, provide evidence for the complex microstructure of brominated tissues of *A. grewingkii* IN teeth. The Br content is approximately equal in EP1 and EP3 but slightly decreases in the intermediate layer EP2, which is exposed at the apical tooth surface (Figures 1I, J, K). It appears that this intermediate layer is less cross-linked (and, therefore, more flexible) and prevents propagation of cracks, such as that shown in Figure 1K, which extends from the external, highly brominated EP layer inside the IN tooth. In our opinion, Br-dependent sclerotization of the organic matrix plays the key role in strengthening the external surfaces of teeth on the cutting edge of *A. grewingkii* mandible.

The concentration of total dissolved solids in the water of Lake Baikal is low, approximately 0.1 g L^−^^1^ [28], and the concentration of bromine is only about 20 ppb, or 2×10^−5^ g L^−^^1^ [15], which is much lower than in seawater [29]. The concentration of this element in bottom sediments varies from 0 to 15 ppm (0.015 g L^−^^1^), peaking during warming periods characterized by active development of the biota [30]. In the bodies of littoral amphipods, Br concentration reaches 300–400 ppm (0.3–0.4 g L^−^^1^), compared to 4–25 ppm (0.004–0.05 g L^−^^1^) in other littoral organisms such as algae, sponges, and caddis worms (S.M. Boiko, unpublished). The content of Br in the EP of *A. grewingkii* IN teeth is three to four orders of magnitude higher than in the bodies of littoral amphipods and six to seven orders of magnitude higher than in the Baikal water. Being polyphagous, this amphipod can accumulate Br both from the food chain and from ingested bottom sediments. Such a high level of Br accumulation and its distinct localization in different EP layers are evidence for the existence of mechanisms of active Br transport formed in the course of adaptive evolution of this species.

In the cuticular “tools” of invertebrates (dactyli, claws, teeth, etc.), which are exposed to high mechanical loads, heavy elements (including Br) account for 1–25% of dry weight [31]–[33]. The concentration of Br in other structural tissues of marine invertebrates also reaches a percent level [31]–[33]. For example, the concentration of halogens in the sclerotized skeleton of different black coral species ranges from 2 to 5% [32]. The main advantage of brominated over calcified cuticle is in its high fracture resistance [11]. The mechanism of change in the mechanical properties of the cuticle upon bromination is unknown, although it is supposed that Br induces cross-linking between polypeptide chains in its structural tissues [11]. The contents of Br in marine crustaceans tend to be increased, compared to those in some other invertebrates [29]. The Br-rich cuticle on the spoonlike chelipeds and dactyli of walking legs in many crabs is distinguished by its amber color and transparency [11], [33], [34]. The tips of the dactyli of gnathopods in *A. grewingkii* are also enriched with Br and goldish amber in color, though much paler than IN teeth. Some specialists attribute the characteristic coloration and transparency of cuticular tools to the presence of organic tannins [20], [35], [36].

Thus, we have used a variety of methods to study the morphology and fine structure of MD and the distribution of elements on the surface and sections of IN teeth in *A. grewingkii*, an abundant amphipod species from Lake Baikal. This allowed us to reveal mechanisms for the strengthening of the MD cutting edge that are more complex than those evolved in marine copepods, in which siliceous teeth are underlain by chitin layers [5]. Functional adaptations of MD in polyphagous *A. grewingkii* are aimed at maintaining an optimum balance between mechanical hardness and fracture resistance, which is achieved due to the complex structure and composition of cutting mouthparts. The surface of IN teeth is covered by a very hard siliceous film, which is underlain by softer but more fracture-resistant layers of Br-enriched epicuticle, and the internal tooth tissues are reinforced with Ca salts. Therefore, the MD cutting edge is a complex biocomposite material combining different properties in its individual layers, which in the aggregate provide for its effective functioning.

Evolutionary advantage of *A. grewingkii* with strong MD is in its capacity for polyphagy. The enlargement of feeding spectrum at the expense of large and/or hard food objects causes the increase in the number of fragmented diatom valves, crustacean carapaces and others in pellets. The pellets mixing with the substrate improve its feeding quality for both bacteria and small invertebrates-detritophages inhabiting the sediment column and its surface. This aspect together with high activity of *A. grewingkii* in mixing of the upper sediment layer for searching food determines the role of this amphipod in the cycle of the Lake Baikal ecosystem. Thus, mechanisms of MD strength resistance may have not only evolutionary but also biogeochemical aspects.
